# Combining standard clinical methods with PCR showed improved diagnosis of invasive pulmonary aspergillosis in patients with hematological malignancies and prolonged neutropenia

**DOI:** 10.1186/s12879-015-0995-8

**Published:** 2015-07-01

**Authors:** Melinda Paholcsek, Gabor Fidler, Jozsef Konya, Laszlo Rejto, Gabor Mehes, Evelin Bukta, Juergen Loeffler, Sandor Biro

**Affiliations:** Faculty of Medicine, Department of Human Genetics, University of Debrecen, Nagyerdei krt. 98, H-4032 Debrecen, Hungary; Faculty of Medicine, Department of Medical Microbiology, University of Debrecen, Debrecen, Hungary; Faculty of Medicine, Institute of Internal Medicine, University of Debrecen, Debrecen, Hungary; Faculty of Medicine, Institute of Pathology, University of Debrecen, Debrecen, Hungary; Department for Internal Medicine II, University of Wuerzburg Medical Centre, Wuerzburg, Germany

**Keywords:** Invasive pulmonary aspergillosis, Biomarkers, Combination testing, Acute leukemia, Neutropenic fever

## Abstract

**Background:**

We assessed the diagnostic value of standard clinical methods and combined biomarker testing (galactomannan assay and polymerase chain reaction screening) in a prospective case–control study to detect invasive pulmonary aspergillosis in patients with hematological malignancies and prolonged neutropenia.

**Methods:**

In this observational study 162 biomarker analyses were performed on samples from 27 febrile neutropenic episodes. Sera were successively screened for galactomannan antigen and for *Aspergillus fumigatus* specific nucleic acid targets. Furthermore thoracic computed tomography scanning was performed along with bronchoscopy with lavage when clinically indicated. Patients were retrospectively stratified to define a case-group with “proven” or “probable” invasive pulmonary aspergillosis (25.93 %) and a control-group of patients with no evidence for of invasive pulmonary aspergillosis (74.07 %). In 44.44 % of episodes fever ceased in response to antibiotic treatment (group II). Empirical antifungal therapy was administered for episodes with persistent or relapsing fever (group I). 48.15 % of patients died during the study period. Postmortem histology was pursued in 53.85 % of fatalities.

**Results:**

Concordant negative galactomannan and computed tomography supported by a polymerase chain reaction assay were shown to have the highest discriminatory power to exclude invasive pulmonary aspergillosis. Bronchoalveolar lavage was performed in 6 cases of invasive pulmonary aspergillosis and in 15 controls. Although bronchoalveolar lavage proved negative in 93 % of controls it did not detect IPA in 86 % of the cases. Remarkably post mortem histology convincingly supported the presence of *Aspergillus* hyphae in lung tissue from a single case which had consecutive positive polymerase chain reaction assay results but was misdiagnosed by both computed tomography and consistently negative galactomannan assay results. For the galactomannan enzyme-immunoassay the diagnostic odds ratio was 15.33 and for the polymerase chain reaction assay it was 28.67. According to Cohen’s kappa our in-house polymerase chain reaction method showed a fair agreement with the galactomannan immunoassay. Combined analysis of the results from the *Aspergillus* galactomannan enzyme immunoassay together with those generated by our polymerase chain reaction assay led to no misdiagnoses in the control group.

**Conclusion:**

The data from this pilot-study demonstrate that the consideration of standard clinical methods combined with biomarker testing improves the capacity to make early and more accurate diagnostic decisions.

**Electronic supplementary material:**

The online version of this article (doi:10.1186/s12879-015-0995-8) contains supplementary material, which is available to authorized users.

## Background

Patients suffering from hematological malignancies and prolonged neutropenia are significantly affected by life-threatening invasive fungal infections, particularly invasive pulmonary aspergillosis (IPA). Despite improvements in transplantation practices and antifungal therapy the treatment of patients with IPA is still challenging [1]. Diagnosis of IPA is difficult and delayed diagnosis contributes to the increasing severity of IPA associated high morbidity and mortality rates [2, 3]. Standard radiology as thoracic computed tomography (CT) scan and classical mycology of bronchoalveolar lavage (BAL) have low sensitivity and may be no more specific than fevers of unknown origin that are refractory to treatment with broad-spectrum antibiotics; especially in neutropenic patients [4, 5]. Despite many limitations empirical antifungal therapy is regarded as a standard treatment in these patients. The relatively low incidence of IPA is accompanied by the risk of over treating people thus subsequently loading them with unnecessary drug toxicity [6]. On one hand these fever-driven approaches use a very limited diagnostic work-up in the need of avoiding deleterious delay in the initiation of therapy on the other hand they may miss IPA developing in the absence of fever [7]. An alternative management strategy could be preemptive antifungal therapy guided by IPA associated risk factors such as mycological tools, imaging techniques, detection of specific biomarkers by *Aspergillus* galactomannan enzyme immunoassay (GM-EIA) or sensitive polymerase chain reaction (PCR) [7, 8]. The routine use of non-culture based molecular assays might have the potential to fill the gap between over- and under treatment providing improved diagnosis compared to classical methods and single assay detection [9]. The primary aim of this non-randomized study was to assess the applicability of our enhanced patient surveillance strategy in routine clinical practice on our retrospectively stratified patient population. A further aim was to estimate the diagnostic utility of combined biomarker testing from the onset of febrile neutropenia by combining serum GM-EIA screening and *facC*-PCR detection. This could show how many patients would receive a misdiagnosis on the basis of a fever driven empirical approach without regarding results from sensitive and specific biomarker testing.

## Methods

### Study population

Between June 06, 2012 and May 08, 2013, 27 febrile neutropenia episodes (FNEs); 16 males with the median age of 42.5 (range 14–75) and 11 females with the median age of 45.5 (range; 19–66) (Table 1) were included at the onset of febrile neutropenia. The patients had different hematological malignancies (mainly acute leukemia) receiving stem cell transplantation and intensive chemotherapy (neutrophil count < 0.5 × 10^9^ cells/L; temperature > 38 °C of febrile neutropenia recorded twice or > 38.5 °C recorded once) in our local prospective case–control study. The study protocol was approved by the ethics committee of the University Hospitals of Debrecen, Hungary. Written consent was obtained from patients and the caretakers of the children in all of the cases. PCR testing was performed parallel with GM-EIA and needed no ethical approval. PCR results were sent back to clinicians however were not taken into clinical decision making.

**Table 1:** Results of combined biomarker testing of patients with febrile neutropenia^a^ at the University Hospitals of Debrecen^b^

Patient ID	Underlying condition	Sex, age (yr)	EORTC/MSG classification	No. of specimens per episode	No. of GM specimens		No. of PCR specimens		No. of PCR runs	
					Positive	Negative	Positive	Negative	Positive	Negative
1	PTCL	M, 18	possible	8	0	4	1	3	3	9
2	NHL	M, 15	possible	14	0	7	2	5	2	19
3	OM	M, 16	possible	16	0	8	1	7	3	21
4	AML	M, 26	proven	18	0	7	10	1	18	15
5	OM	F, 53	possible	6	1	2	0	3	0	9
6	AML	M, 55	possible	4	0	2	1	1	1	5
7	AML	M, 52	proven	4	2	0	2	0	3	3
8	AML	F, 28	proven	6	1	2	3	0	3	4
9	MM	M, 70	possible	2	0	1	0	1	0	3
10	ALL	F, 27	probable	8	2	0	6	0	13	1
11	OM	M, 57	possible	2	0	1	0	1	0	3
12	OM	F, 66	possible	6	0	3	0	3	0	9
13	AML	F, 55	probable	8	2	2	4	0	8	4
14	AML	F, 46	possible	2	0	1	0	1	0	3
15	MM	F, 44	possible	2	0	1	0	1	0	3
16	OM	M, 56	proven	6	0	3	1	2	1	8
17	HL	F, 26	possible	4	0	2	0	2	0	6
18	AML	F, 50	possible	2	0	1	1	0	3	0
19	ALL	M, 14	possible	4	0	2	0	2	0	6
20	AML	F, 45	possible	6	0	3	0	3	0	9
21	OM	M, 17	possible	6	0	3	0	3	0	9
22	OM	F, 19	possible	8	0	4	3	1	3	9
23	NHL	M, 57	possible	2	0	1	0	1	0	3
24	AML	M, 36	possible	6	1	2	0	3	0	9
25	NHL	M, 75	proven	6	2	1	1	2	1	8
26	AML	M, 49	possible	4	0	2	0	2	0	6
27	OM	M, 16	possible	2	0	1	0	1	0	3
			Total:	162	77		85		249	

^a^Neutrophil count < 0,5 × 10^9^ cells/L; ^b^University of Debrecen, Institute of Internal Medicine; *AML* Acute Myeloid Leukemia, *ALL* Acute Lymphoblastic Leukemia, *MM* Myeloma Multiplex, *NHL* Non-Hodgkin Lymphoma, *PTCL* Peripheral T-cell Lymphoma, *OMs* other malignancies, *P* present, *A* absent, *NP* not performed, *CT* thoracic computed tomography, *BAL* bronchoscopy with lavage

### Search strategy

For FNE, combined biomarker testing (CBT) was commenced. Serum samples were prospectively screened for the presence of genus and species specific biomarkers galactomannan (GM) and *facC* genes. The results were anonymously evaluated [10]. Screen-ing for GM-antigenemia Platelia *Aspergillus* (GM-EIA) was used on the basis of the OD_450/620_ ≧ 0.5 cutoff value.

The real time PCR detection of *A. fumigatus* specific DNA was based on the *Streptomyces factor C* orthologous gene (*facC*-PCR): this was most likely horizontally transferred to a few filamentous fungi including *A. fumigatus* as previously described [11]. Since the *facC* gene that was acquired by horizontal gene transfer [12] is only present in a few fungal species [13] and they diverged millions of years ago, we were able to design species-specific assays that do not cross-react even with closely related species that carry this gene therefore the analytical specificity is increased as compared to other assays. At the onset of fever broad-spectrum antibiotic treatment was administered according to the published Infectious Diseases Society of America guidelines [14]. Furthermore standard chest CT and BAL analysis were performed as clinically indicated.

### Antifungal therapy

Persistent fever, positive GM assay along with either positive CT and/or positive BAL was considered sufficient evidence to initiate broad spectrum antifungal treatment. In the case of fever that ceased after 48 h of antibiotic treatment, with negative CT and/or BAL results (when performed), we suggested extending the surveillance period. These cases were monitored for the presence of *Aspergillus* specific biomarkers (GM and the *facC-*orthologous gene) in parallel with thorough clinical examinations on a weekly basis.

### Stratification of febrile neutropenia episodes

Cases were retrospectively stratified according to revised European Organization for the Research and Treatment of Cancer/Mycosis Study Group (EORTC/MSG) criteria in order to define a case-group of “proven” or “probable” IPA (7 patients) and a control-group possible IPA (20 patients with no EORTC/MSG evidence of invasive aspergillosis) [10, 15].

### Ascertainment bias

Ascertainment bias can potentially overestimate the diagnostic odds ratio (DOR) for the benefit of the gold-standard GM-EIA method. By introducing false-positives it can erode the discriminatory power of the other diagnostic methods (e.g. *facC-*PCR).

### Postmortem histology

The specificity of *Aspergillus* infection morphology was critically addressed in the histological samples obtained following autopsy. Samples were taken from the major organs according to a standard protocol, but no macroscopic signs of organ involvement were seen other than in the lungs. Lung sampling was performed from three independent parts of the potentially infiltrated lung parenchyma. Histological evaluation clearly proved the existence of invasive fungal infection. The detailed feature of the PAS and/or Hematoxylin and Eosin (H&E) stained hyphae supported the *Aspergillus* specific PCR assay based assumption of invasive aspergillosis in the lung although other filamentous fungi may resemble *Aspergillus* hyphae therefore definite identification is not possible on a morphological basis.

### Serum specimens

As part of our combined biomarker testing [9] consecutive serum samples (3 × 3 ml) were routinely drawn from patients at the onset of febrile neutropenia and sent to the Department of Medical Microbiology, Debrecen, Hungary for GM antigen monitoring. The GM assays required 300 μl of serum samples and the rest was stored at −20 °C. The two other intact serum tubes were forwarded to the Department of Human Genetics, Debrecen, Hungary for *facC*-PCR and opened only in our laboratory.

### DNA extraction

All DNA extraction steps were performed in a class II laminar-flow cabinet to avoid environmental contamination. Serum samples obtained from healthy volunteers were pooled and screened for *Aspergillus fumigatus* nucleic acid contamination prior to use as ex-traction negative controls (2.5 ml). In order to control the efficiency of the fungal nucleic acid extraction and potential PCR inhibition the QuantiFast Pathogen PCR+IC Kit (Qiagen, Hilden, Germany) was used to prepare extraction positive control samples (2.5 ml) containing 10 μl of internal control DNA. 2.5 ml of serum samples obtained from episodes were extracted along with the extraction positive and negative controls using the High Pure Viral Nucleic Acid Large Volume Kit (Roche Applied Science) according to manufacturer’s instructions. The elution volume was adjusted to 45 μl. 12 μl of eluate was used in each PCR reactions.

### *FacC-*PCR based real-time detection

Species specific hydrolysis assays were used that targeted the *facC* orthologous genes present in *A. fumigatus* [11]. *A. fumigatus* specific duplex TaqMan-LNA hydrolysis assay was used targeting *facC* orthologous genes (AFUA_3G14910, and AFUA_5G00540) present in two copies (on Chr#3 and on Chr#5 respectively) in *A. fumigatus* genome. The duplex assay contained AFUA_3G14910, and AFUA_5G00540 specific primers (0.8 μM for each) and probes (0.5 μM for each). The primers and probe sequences were Afu3_Fw (5′-CTTTGGCATCGCGACAGT-3′), Afu3_Rev (5′-GAGGGGGCAGAGAGATC-3′), Afu3_probe (5′-FAM-CAGCATCC-DQD-3′, where DQD is dark quencher dye), Afu5_Fw (5′-CGTCCAACAGTCATTCACCTT-3′), Afu5_Rev (5′-ATCGCGACGGTTTACGAT-3′), Afu5_probe (5′-FAM-CCGCCGCC-DQD-3′). Amplification was carried out on LightCycler® 2.0 Carousel-Based platform (Roche Applied Science). 5× LightCycler TaqMan Master (Roche Applied Science) was used according to manufacturer’s instructions containing Uracil-DNA Glycosylase for carryover prevention during PCR and the reaction volumes were settled to 20 μl in glass capillaries where 12 μl of elutes were monitored for the presence of *A. fumigatus* genomic DNA. Each real-time run contained template-positive (PCR-IC spiked with 100–1000 GE *Aspergillus fumigatus* gDNA) and template-negative (PCR-NTC containing PCR grade nuclease free water) control samples.

## Data interpretation

### Discriminatory power of standard methods

The discriminatory power of the CT scans combined with either GM-EIA or with BAL were analyzed by comparison with patient status (cases and controls), which allowed the negative predictive values (PV^−^) to be calculated.

(i) **Real time*FacC*assays.** In this study triplicate *facC*-PCR reactions were performed. Single positive assay result from the triplicate reactions was used to define a sample as PCR positive [16–18].
(ii) **Galactomannan ELISA.** The Platelia *Aspergillus* GM-EIA (Bio-Rad Laboratories, Hungary) was used for galactomannan screening. GM-EIA cutoff values were determined using the OD_450/620_ value of sample/OD_450/620_ value of control. While evaluating single specimens any value above the OD_450/620_ ≧ 0.5 cutoff value was considered positive as requested for *in vitro* testing. While evaluating episode GM-EIA statuses the ratio of positive versus negative results were analyzed. In the case of equality, an episode was considered positive.

### Statistical analysis of CBT

Along with reporting of descriptive statistics for categorical data; median, mean, standard deviation (±SD), diagnostic performance parameters (by doing the 2×2 contingency tables of cases and controls) with discriminatory properties of post-test probabilities (likelihood ratios) and accuracy indices (DORs) were calculated with 95 % confidence intervals (95 % CI) for GM screening (GM-EIA) and for the nucleic acid based diagnostic method (*facC-*PCR) respectively.

### Biomarker measurement comparison

In this study 162 biomarker analyses from 27 febrile neutropenic episodes with the mean number of 6 ± 4.18 specimen/episodes (range; 2–18) were performed. For assessing sample concordance between the established gold-standard GM-EIA screening and our in-house *facC*-PCR method inter annotator agreement was calculated using the Cohen’s kappa test where values greater than 0.8 represent an *excellent* agreement, values of 0.61–0.8 represent a *substantial* agreement, values of 0.41–0.6 a *moderate* agreement, values of 0.21–0.4 a *fair* agreement and below 0.20 a *slight* agreement between two classifiers.

### ROC analysis

To measure the diagnostic accuracy of the results from GM-EIA and *facC*-PCR testing were converted to qualitative (positive, negative) indexes. In the case of *facC-*PCR every single run was evaluated independently. After calculating the series of sensitivity (Se) and specificity (Sp) reports at every single decision threshold the receiver operating characteristic (ROC) curve was edited by plotting the true positive values (sensitivity values on y-axis) versus the false positive rates (1-specificity values on x-axis). Area under the ROC curve (AUC) was estimated with 95 % confidence intervals (95 % CIs) and with standard errors (±SD) where values greater than 0.91 represent *excellent* discriminatory accuracy, values of 0.81 - 0.9 represent *good* accuracy, values of 0.71 - 0.8 *fair* accuracy, values of 0.61 - 0.7 *poor* accuracy and values of 0.5 - 0.6 represent a diagnostically *not useful* test. Fisher’s exact test was used to generate two-sided *P* values with a *P* value of < 0.05 being considered significant. Furthermore likelihood ratios (LR+, LR-) and predictive values (PV+, PV-) were estimated.

## Results

### Classification of febrile episodes

Patient demographics with regard to EORTC/MSG classification are shown in Table 1.

### Discriminatory power achieved by combining CT with GM-EIA and BAL

**Fig. 1:**
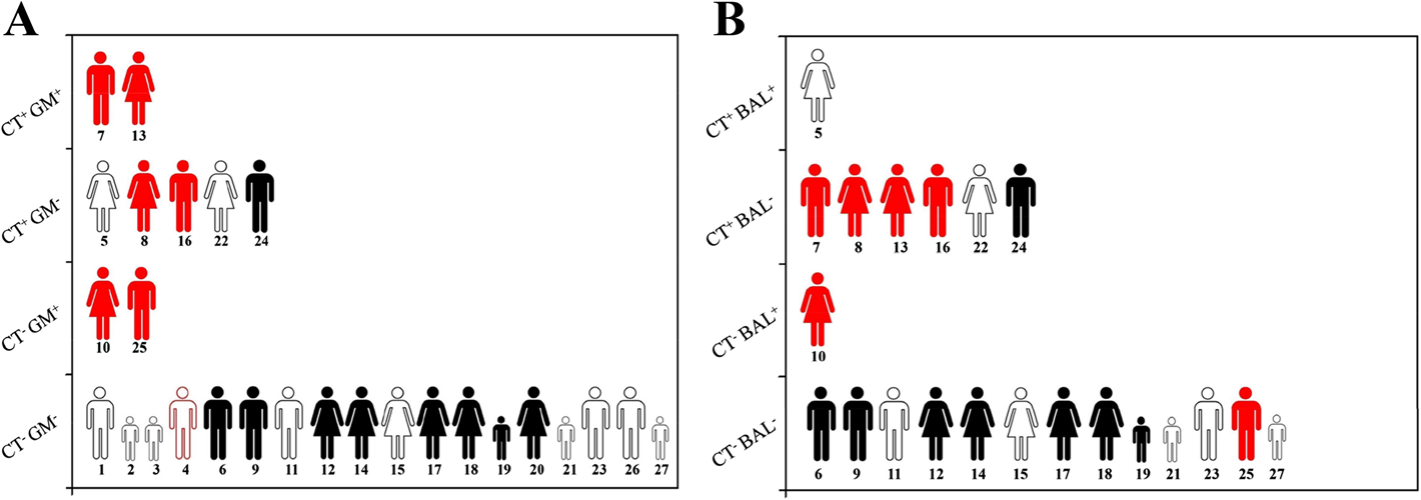
Representation of the discriminatory power of *Aspergillus* GM-EIA (**a**) and BAL (**b**) when combining these with chest CT. **a** CT^+^-GM^+^; episodes proved to be positive when testing both by CT and *Aspergillus* GM-EIA, CT^+^-GM^−^; episodes proved to be positive when scanning by CT and negative when testing by *Aspergillus* GM-EIA, CT^−^-GM^+^; episodes proved to be negative when scanning by CT and positive when testing by *Aspergillus* GM-EIA, PCR^−^-GM^−^; episodes proved to be negative when testing by both CT and *Aspergillus* GM-EIA. Black symbols represent controls while red symbols represent cases. Contoured symbols represent episodes with ceased fever, while shaded symbols represent episodes with present or recurrent fever refractory to broad spectrum antibiotic treatment. Small sized symbols represent children. Numbers denote patient ID numbers. **b** CT^+^-BAL^+^; episodes proved to be positive when testing both by CT scanning and BAL, CT^+^-BAL^−^; episodes proved to be positive when testing by thoracic CT and negative when testing by BAL, CT^−^-BAL^+^; episodes proved to be negative when testing by thoracic CT and positive when testing by BAL, CT^−^-BAL^−^; episodes proved to be negative when testing both by thoracic CT and BAL. Black colored symbols represent controls while red colored symbols represent cases. Contoured symbols represent episodes with ceased fever, while shaded symbols represent episodes with present or recurrent fever refractory to broad spectrum antibiotic treatment. Small sized symbols represent children. Numbers denote patient ID numbers

Concordant negative GM-EIA together with thoracic CT scans has been shown to have the highest discriminatory power to exclude IPA as a diagnosis. The calculated PV^−^ proved to be high; 0.94 (17 controls, 1 case) (Fig. 1a). When combining BAL with thoracic CT scans the calculated PV^−^ proved to be; 0.92 (12 controls, 1 case). BAL was performed in 77 % of all episodes. (6 cases and 15 controls). In the control group BAL excluded IPA in 93 % of the episodes (14 of 15). BAL was able to diagnose IPA in one case only (patient ID 10). In this case CT failed to detect IPA (Fig. 1b).

### Diagnostic performance of the biomarker assays

Performance parameters such as sensitivity (Se), specificity (Sp), likelihood ratios (LR^+^, LR^−^), predictive values (PV^+^, PV^−^) and diagnostic odds ratio (DOR) were estimated for GM-EIA serology and *facC*-PCR by using the current cutoff index for the gold-standard GM-EIA (OD_450/620_≧0.5) and by considering a single positive PCR run as significant for *facC-*PCR. Performance parameters with and without stratification correction are shown in Table 2.

**Table 2:** Diagnostic performance of GM-ELISA and *facC-*PCR when testing serum samples

Parameter	Platelia *Aspergillus*-EIA	*facC-*PCR
% Se (95 % CI)	52,63 (28,90-75,51)	69,23 (48,21-85,63)
% Sp (95 % CI)	98,31 (90,88-99,72)	68,85 (55,71-80,09)
LR+	31,05	2,22
LR-	0,48	0,45
PV+ %	90,91	48,65
PV- %	86,57	84,00
DOR (95 % CI)	64,44 (7,342-565,694)	4,974 (1,81-13,434)
**% Se (95 % CI)**	**37,5 (17,97-57,48)**	**84 (65,46-93,19)**
**% Sp (95 % CI)**	**96 (87,02-99,54)**	**83,02 (70,19-91,91)**
**LR+**	**9,5491**	**4,85**
**LR-**	**0,6651**	**0,21**
**PV+ %**	**89,4268**	**75,68**
**PV- %**	**62,9298**	**88,00**
**DOR (95 % CI)**	**15,33 (3,117-75,420)**	**28,667 (9,048-90,823)**
***P*** **value**	***P*** **< 0,0001**	***P*** **< 0,0001**

Calculated diagnostic performance for Platelia *Aspergillus GM-*EIA and *facC-*PCR at the standard cutoff OD450/620≧0.5 for GM-EIA and by considering ≧1 positive Cq as significant/triplicate. Performance parameters calculated after stratification corrections based on autopsy data are highlighted with bold letters. *Se* sensitivity, *Sp* specificity, *95 % CI* 95 % confidence interval, *LR+* likelihood ratio positive, *LR-* likelihood ratio negative, DOR, *PV+* positive predictive value, *PV-* negative predictive values

### Data on CBT

**Fig. 2:**
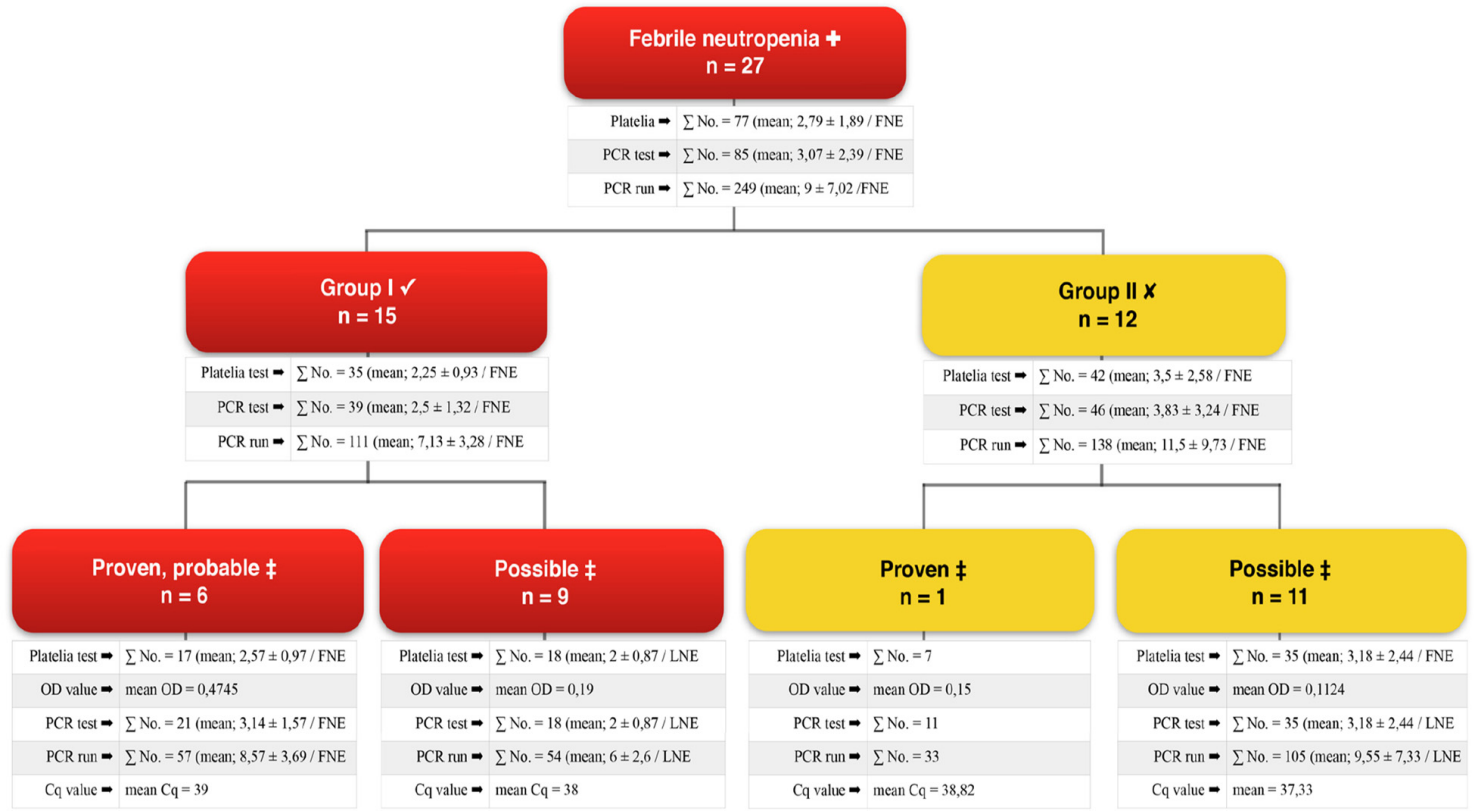
Data on CBT in fevered neutropenia episodes at the University Hospitals of Debrecen. (^✤^) Neutrophil count less than 500/mm^3^. (^✔^) persistent fever for more than 96 h, refractory to antibiotic treatment, (group I patients); (^✘^) lack of persistent fever more than 96 h, (group II patients); (^‡^) according to European Organization for Research and Treatment of Cancer criteria. (FNE); febrile neutropenia episode. There were in total 162 tests (mean; 2.93 ± 2.14/FNE) performed. 77 serum specimens (mean; 2.79 ± 1.89/FNE) were screened for the presence of GM antigen and 85 PCRs (mean; 3,07 ± 2.39/FNE) were performed supported by 249 runs (mean; 9 ± 7.02/FNE) and targeting special fungal nucleic acid marker genes (*facC*-PCR) of prokaryotic origin. Reporting of descriptivce statistics with mean values and standard error (±SE) are shown

On completion of combined biomarker testing categorical data; median, mean, standard deviations (±SD) were calculated for GM screening (GM-EIA) and for the nucleic acid based diagnostic method (*facC-*PCR). Data are shown in Fig. 2.

### Sample concordance

Sample concordance was assessed between the two classifiers; Platelia *Aspergillus* GM-EIA and facC-PCR showing a fair agreement generating an observed ratio of 70.13 % (agreement; 54 of 77) and a kappa statistic of 0.258 (95 % confidence interval from 0.057 to 0.460). Distributions of the positive and negative hits (77 GM-EIA OD_450/620_ values, 85 PCR Cq-s) were visualized by dot histogram (Additional file 1: Figure S1).

### Diagnostic accuracy

**Fig. 3:**
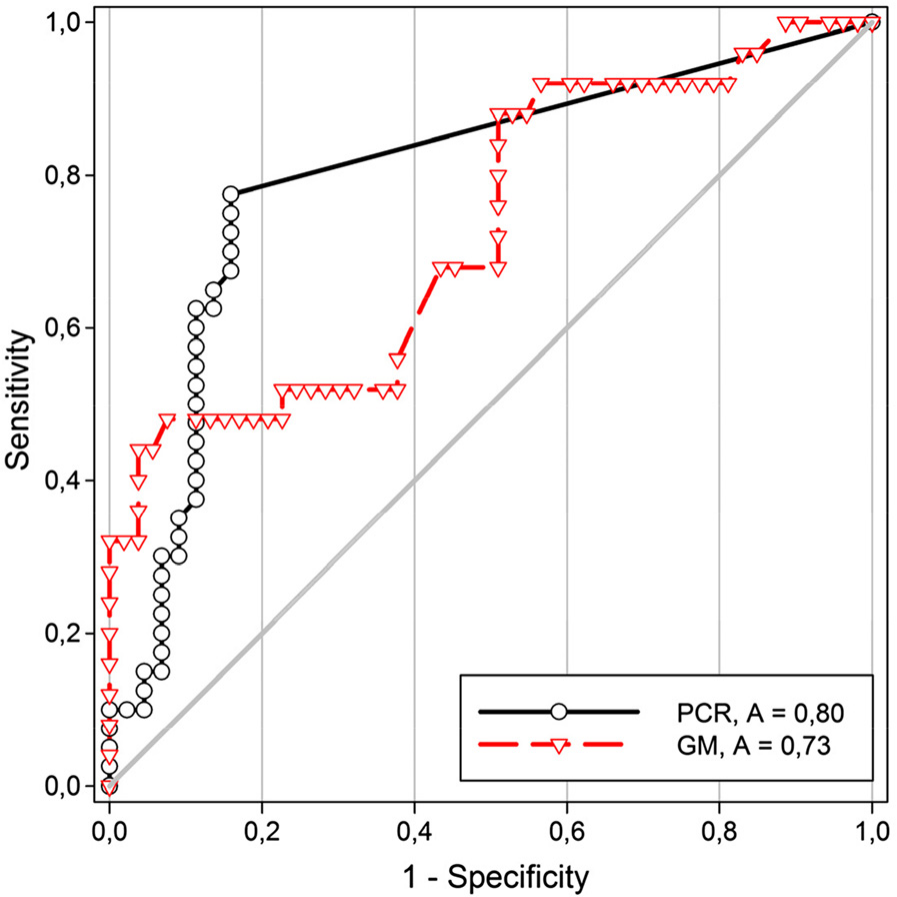
Diagnostic performance (receiver operating characteristic curves) for *Aspergillus* GM-EIA and *facC-*PCR methods. ROC curve illustrating the diagnostic performance of GM-EIA and *facC*-PCR at different discriminatory thresholds by plotting true positives (sensitivity) against false positives (1-specificty)

Area under the ROC curve (AUC) was estimated for both classifiers; GM-EIA and *facC*-PCR. Values represent for GM-EIA a *fair* while for *facC-*PCR a *good* discriminatory property. The area under the ROC-curve (AUC) for GM-EIA was 0.7283 (95 % CI 0.6031-0.8535); *P* value of < 0.0012 ± 0.0639 while that for the *facC-*PCR was 0.8004 (95 % CI 0.6976-0.9031); *P* value of < 0.0001 ± 0.05243. ROC curves with the calculated AUCs are shown in (Fig. 3).

### Combined biomarker assay performance by section analysis

**Fig. 4:**
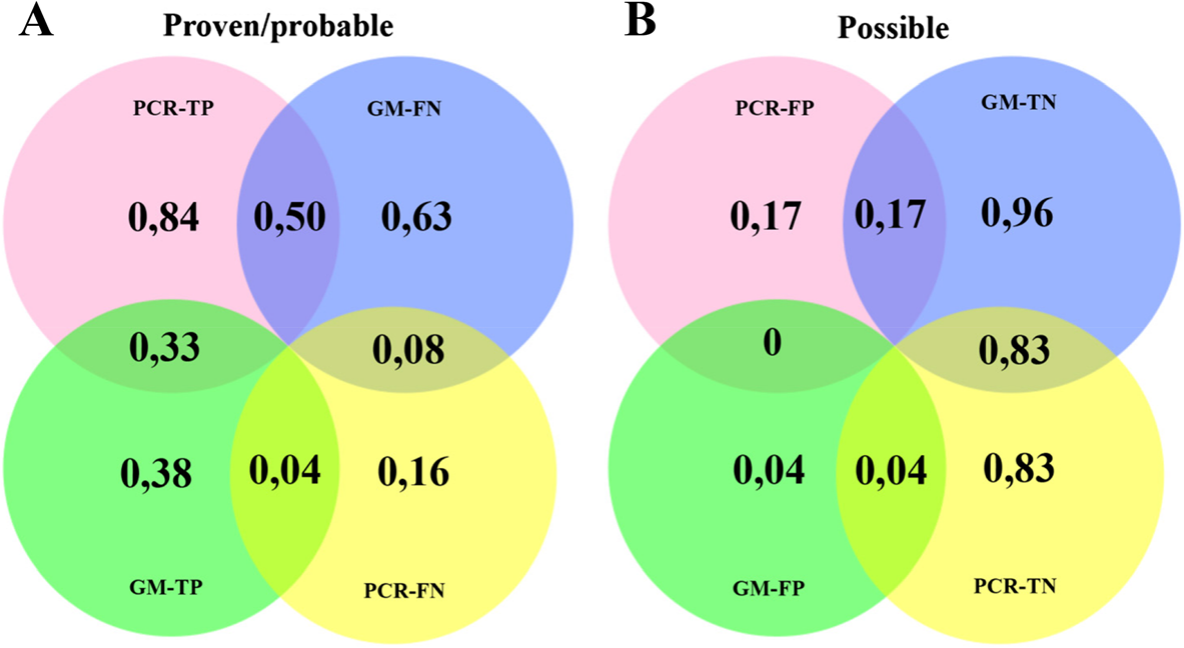
Venn-diagrams with section-analysis comparing the outcomes of Platelia *Aspergillus* GM-EIA and *facC-*PCR. *TP* true-positive, *FN* false-negative, *TN* true-negative, *FP* false-positive. Cases (proven and probable IA) with positive GM-EIA and PCR results were regarded as true positive (TP) while those with negative outcomes were considered to be false negative (FN). Controls (unclassified patients with no EORTC/MSG evidence of IA) with negative GM-EIA and PCR results were coded to true negative (TN) and those with positive outcomes false positive (FP). **a** 27 of 32 (84 %) and 9 of 24 (38 %) serum samples from cases (proven/probable) proved to be true positive (TP) when testing with *facC-*PCR and GM-EIA respectively. GM-EIA failed to detect 15 of 24 and *facC*-PCR 5 of 32 samples. **Section analysis:** 8 of 24 specimens from cases (33 %) proved to be true positive (TP) with both assays and 3 of 24 (8 %) found to be negative (FN) by both of them. There was only one specimen (4 %) of 24 that was missed by *facC*-PCR but not by GM-EIA. 50 % of specimens (12 of 24) however were detected only by PCR but not with GM-EIA. **b** 51 of 53 (96 %) and 44 of 53 (83 %) serum specimens from controls (unclassified patients with no evidence of IA) proved to be negative (TN) thus 2 of 53 (3,8 %) and 9 of 53 (17 %) were undetected. There were 9 of 53 (17 %) specimens that proved to be false-positive with PCR but real-negative with GM and only 2 of 53 (4 %) that proved to be false-positive with GM-EIA but true-negative with *facC*-PCR. **Section analysis:** 83 % of specimens (44 of 53) remained consistently negative with both of the assays but there were none that proved to be false-positive when testing with both methods

Testing control serum specimens with GM-EIA and *facC*-PCR yielded a high specificity (96 %) (Fig. 4b). Only two control specimens from episodes with no EORTC/MSG evidence of IPA were false-positive when testing with GM-EIA. GM-EIA failed to detect the presence of *Aspergillus* GM antigen in 63 % of the cases and sensitivity proved to be only 38 % (Fig. 4a). In the case population *facC-*PCR was able to detect the presence of *Aspergillus fumigatus* specific nucleic acid target molecules in 84 % of cases by considering a single positive run from triplicate reactions as significant. Conversely *facC-*PCR failed to detect 16 % of the samples from cases (Fig. 4a). With regard to specificity, 83 % of control specimens were negative when tested with *facC-*PCR ensuring a relatively high false-positivity rate of 17 % for PCR (Fig. 4b). Comparison analysis was done by examining the different sections of Venn-diagrams shown in Fig. 4. Only paired results were considered and those that were superior in numbers (in the cases of patient ID 4 and ID 10 for the benefit of *facC*-PCR) were excluded from the section analysis. In doing so we can see that 50 % of specimens from cases were detected only by *facC*-PCR but not with GM-EIA thus 8 % of specimens from cases were missed with either of the two methods. It is noteworthy that in 83 % of all cases when testing control specimens (from episodes with no EORTC/MSG evidence of IPA) both assays remained consistently negative furthermore there were no false-positive cases when testing with both methods.

### Survival and mortality data

The overall mortality rate was 48.14 % (Additional file 2: Figure S2). 13 of 27 patients died during the surveillance period; 4 of 13 were cases (30.77 %) and 9 were controls (69.23 %). Post mortem histology (PMH) was pursued in 53.85 % (7 of 13 patients) of fatalities. 10 of 23 people (43.47 %) with patient ID 4, 9, 11, 14, 15, 17, 18, 19, 22, 24 (Fig. 1a) from the GM-EIA negative group and 3 of 4 people (75 %) with patient ID 7, 10, 13 from the GM-EIA positive (Fig. 1a) group. In the case of one episode of the latter group PMH proved to be negative (Additional file 2: Figure S2; patient ID 10).

### Stratification correction at autopsy

In two cases major discrepancies were observed between the clinical and postmortem findings.

(i) Autopsy proved hyphal tissue invasion in only one patient (Additional file 2: Figure S2; patient ID 4). Interestingly this patient’s fever ceased in response to antibiotic treatment and provided negative CT result along with 7 consecutive negative GM-EIA tests but generated positive PCR results in 10/11 runs (Fig. 4). This case was thus classified “possible” and remained undiagnosed. It was only at autopsy that this patient was confirmed to have had IPA. The presence of fungal hyphal invasion in the lung tissue was confirmed by PAS and H&E staining and the episode was subsequently classified “proven” (Additional file 3: Figure S3).
(ii) One episode (patient ID 24) was originally classified “probable” based on persistent fever refractory to broad spectrum antibiotic treatment, positive thoracic CT, and one positive GM-EIA result out of three but definite negative PCRs (9 of 9 runs). As postmortem histology could not demonstrate fungal involvement, episode was reclassified “possible” (Additional file 4: Figure S4).

## Discussion

The incidence of life threatening invasive fungal infections (IFI) caused by filamentous fungi is continuously increasing [19, 20]. *Aspergillus* species are among the most common human pathogens causing serious infections in severely immunocompromised patients and those suffering from prolonged neutropenia [21, 22]. Patients with acute myeloid leukemia (AML) are considered to be at the highest risk for developing neutropenia and consequent IFI [23]. Adequate antifungal therapy initiated early in the course of disease could mitigate devastating effects of IFI. Despite advances in the field of antifungal therapy and improvements in diagnosis through the use of rapid methods to screen for *Aspergillus* genus (GM by sandwich ELISA) or species specific (nucleic acid detection by PCR) biomarkers, estimation of the risk of infection has remained difficult thus clinicians rely on empirical treatment. The limitations of conventional diagnostic methods such as cultures and radiology contribute to delayed treatment of disease. IPA occurs mainly among people with prolonged neutropenia, and only few data are available on IPA in non-neutropenic patients [24]. Our prospective study introduces a combined biomarker monitoring strategy for the management of IPA in patient groups developing neutropenia by using a nucleic acid based real-time diagnostic adjunct to more established diagnostic techniques to enable early and more accurate diagnostic decisions.

An aspergillosis surveillance group was established at the University Hospitals of Debrecen comprising geneticists, molecular biologists, microbiologists and physicians, to assess the discriminatory power of combined biomarker testing by considering *facC*-PCR results together with those of the GM-EIA in our local patient population including patients with different hematological malignancies. The study was observational, thus no diagnostic or treatment decisions were based on the study’s results. According to our inclusion criteria patients with febrile neutropenia were considered from the appearance of fever. FNEs underwent combined biomarker testing by screening of sera for the presence of GM antigenemia (GM-EIA) and the *facC*-PCR with the mean duration of 3.11 ± 2.36 days.

During patient surveillance thoracic CT scans were performed on FNEs and IPA was confirmed in 57 % (4 of 7) of cases; 3 episodes with proven and one episode with probable IPA thus generating a low PV^+^ of 0.57 but a relatively high PV^−^ of 0.85. In one case of a patient (ID 16) with persistent fever, clinical diagnosis of invasive mold infection (IMI) was supported by CT even though BAL culture and GM-EIA (3/3) were consistently negative. Bronchoscopy with lavage was performed in 78 % of episodes (20 of 27 patients), which yielded an extremely low PV^+^ (0.16). False positivity was observed in only one case (patient ID 5) ensuring a high PV^−^ (0.93). Interestingly this patient provided misleading CT results and gave a positive (1/3) GM-EIA result.

It was noticeable that co-analyzing the GM-EIA results with that of CT scans (Fig. 1) provided sufficient strong discriminatory power to exclude IPA (PV^−^ was 0.94). There was only one case (patient ID 4) with a fever that ceased that proved to be false-negative with both of the primary diagnostic methods (GM-EIA and CT). Analysis of BAL was also negative in this case and the patient did not receive antifungal treatment. However during the extended follow-up period *facC*-PCR showed consecutive positive results and a high PV^+^ of 0.91. In this episode we extended the biomarker surveillance period to 17 days gathering 11 *facC*-PCR and 7 GM-EIA samples which gave contradictory data; 10 positive *facC*-PCRs compared to 7 negative GM-EIA.

PMH was pursued in 7 cases and 2 episodes were reclassified after autopsy (Table 2). In both cases *facC*-PCR convincingly supported the presence (patient ID 4) and the absence (patient ID 24) of *Aspergillus* hyphae. By comparing our in-house *facC*-PCR method with GM-EIA it turned out, that sensitive PCR methods could have the capacity to fill the gaps left behind by serology and CT when co-analyzing their results thus generating specific results with high PV^−^.

## Conclusion

In this pilot-study we have shown the potential of combination testing to diagnose IPA more accurately in FNEs with different hematological malignancies. CBT should combine the gold-standard serology (GM-EIA) with sensitive and specific nucleic acid based techniques (e.g. *facC*-PCR) from the onset of febrile neutropenia. This study is relatively small and carried out over a single year but the observed disease prevalence proved to be high (25.9 %). The sensitivity of GM-EIA was found to be only 36 %, which is lagging behind the average results found in other studies. Although the low number of patients presents the major obstacle to drawing strong conclusions we intend to alert clinicians and researchers in this field to the discriminatory power of combined biomarker testing. The CBT approach considers the results of PCR assays together with the results of GM-serology and radiology in clinical decision making thus complementing the relatively low sensitivity of GM-EIA by the more efficient nucleic acid based methods [25]. In doing so we concluded that our patient management strategy has the potential to be accurate and prompt enough, possibly decreasing hospital expenditures and supporting evidence-based diagnosis.

## Additional files

Additional file 1: Figure S1. Dot histogram (**1/A**) visualizing the distribution of the Platelia *Aspergillus* GM-EIA hits (OD_450/620_) with the regard to patient status. Dot histogram (**1/B**) visualizing the distribution of the *facC*-PCR hits (Cq values) with the regard to patient status. Group-control Cq-values correspond to outcomes by testing specimens from possible episodes while group-case Cq-values correspond to outcomes by testing specimens from proven/probable cases. Additional file 2: Figure S2. Representation of the data on postmortem histology. (IA^+^) the presence of hyphae representative of invasive *fungal* infection was confirmed by PAS or H&E staining. (IA^-^) the presence of hyphae could not be confirmed by PAS or H&E staining. Black symbols represent controls while red symbols represent cases. Contoured symbols represent episodes where fever ceased, while shaded symbols represent episodes with present or recurrent fever refractory to broad spectrum antibiotic treatment. Small sized symbols represent children. Numbers intend to denote patient ID numbers. Additional file 3: Figure S3. Postmortem histological findings from case ID 4 showing congestion and focal atelectasis of the lung (3A, ×10 magnification, H&E staining). Detailed evaluation underlined the *Aspergillus* specific PCR assay based assumption of the presence of invasive *Aspergillus* hyphae (3B). This was also supported by PAS staining (3C, ×40 magnification) although definite identification is not possible on a morphological basis therefore the presence of other filamentous fungi cannot be excluded. Additional file 4: Figure S4. Gross view (**4/A**, ×10 magnification) and high-power image (**4/B**, ×40 magnification) of the postmortem lung specimens in case ID 24. Atelectasis with fibrinohemorrhagic exudate and alveolar damage is clearly visible but evidence of microscopic fungal manifestation is absent.

## Abbreviations

±SD: Standard deviation
AUC: Area under the ROC curve
BAL: Bronchoscopy with lavage
CBT: Combined biomarker testing
CI: Confidence interval
CT: Thoracic computed tomography
DOR: Diagnostic odds ratio
EORTC/MSG: European Organization for Research and Treatment of Cancer/Mycosis Study Group
*facC*-PCR: Factor C PCR
FN: False-negative
FNE: Febrile neutropenic episode
FP: False-positive
GM: Galactomannan
GM-EIA: GM enzyme-immunoassay
H&E: Hematoxylin and Eosin
IA: Invasive aspergillosis
IPA: Invasive pulmonary aspergillosis
LR^−^: Likelihood ratio negative
LR^+^: Likelihood ratio positive
OD: Optical density
PAS: Periodic Acid-Schiff
patient ID number: Patient identification number
PCR: Polymerase chain reaction
PMH: Postmortem histology
P-score: Penalty score
PV^−^: Predictive value negative
PV^+^: Predictive value positive
ROC: Receiver operator curve
TN: True-negative
TP: True-positive
IFI: Invasive fungal infection
