# Supplementary material for: Combining standard clinical methods with PCR showed improved diagnosis of invasive pulmonary aspergillosis in patients with hematological malignancies and prolonged neutropenia
Source: BMC Infect Dis. 2015 Jul 1;15:251. doi: 10.1186/s12879-015-0995-8 (PMC4487853; doi:10.1186/s12879-015-0995-8)

**Figure S1**

Dot histogram (**1/A**) visualizing the distribution of the Platelia *Aspergillus* GM-EIA hits (OD_450/620_) with the regard to patient status. Dot histogram (**1/B**) visualizing the distribution of the *facC*-PCR hits (Cq values) with the regard to patient status. Group-control Cq-values correspond to outcomes by testing specimens from possible episodes while group-case Cq-values correspond to outcomes by testing specimens from proven/probable cases.

**Figure S1/A**

**Figure S1/B**


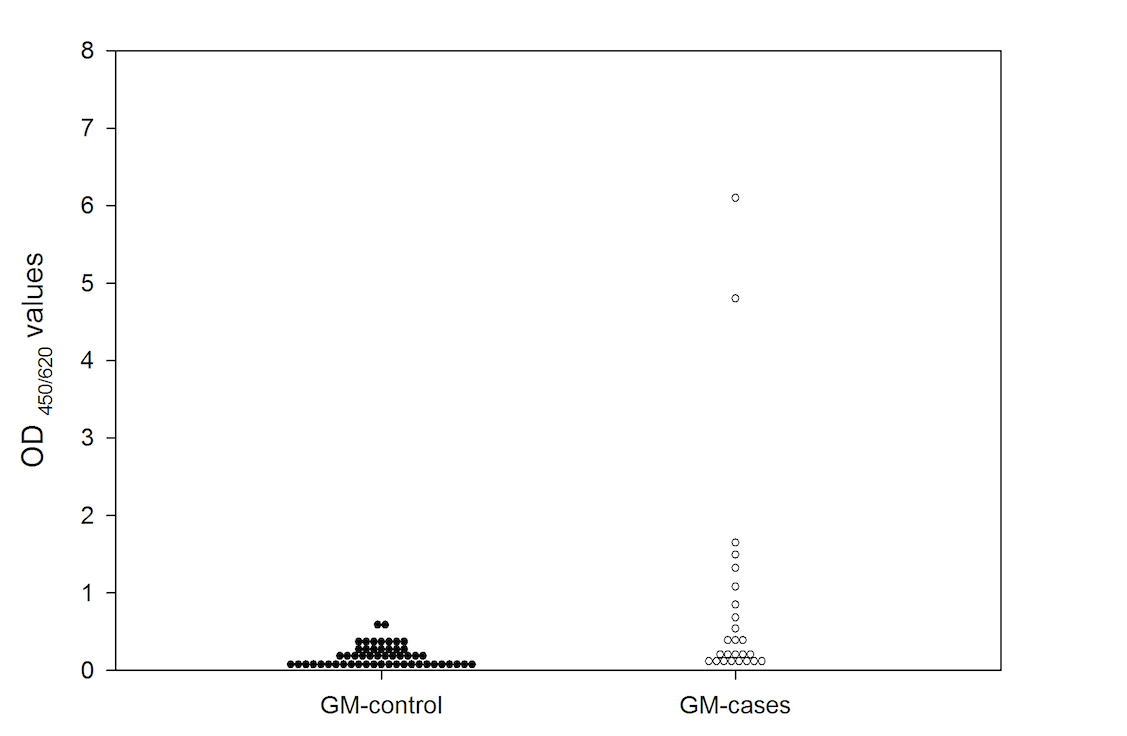

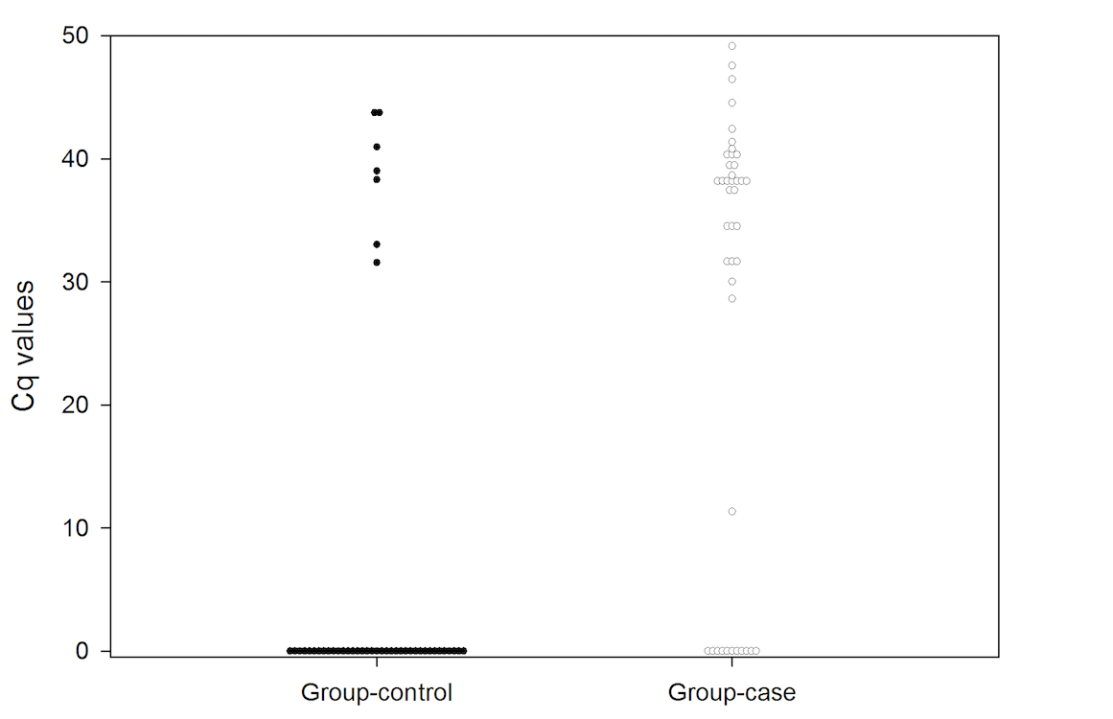

Supplement: Additional file 1: Figure S1. — Dot histogram (1/A) visualizing the distribution of the Platelia Aspergillus GM-EIA hits (OD450/620) with the regard to patient status. Dot histogram (1/B) visualizing the distribution of the facC-PCR hits (Cq values) with the regard to patient status. Group-control Cq-values correspond to outcomes by testing specimens from possible episodes while group-case Cq-values correspond to outcomes by testing specimens from proven/probable cases. [file 12879_2015_995_MOESM1_ESM.docx]
