# Supplementary material for: Combining standard clinical methods with PCR showed improved diagnosis of invasive pulmonary aspergillosis in patients with hematological malignancies and prolonged neutropenia
Source: BMC Infect Dis. 2015 Jul 1;15:251. doi: 10.1186/s12879-015-0995-8 (PMC4487853; doi:10.1186/s12879-015-0995-8)
Supplement: Additional file 2: Figure S2. — Representation of the data on postmortem histology. (IA+) the presence of hyphae representative of invasive fungal infection was confirmed by PAS or H&E staining. (IA-) the presence of hyphae could not be confirmed by PAS or H&E staining. Black symbols represent controls while red symbols represent cases. Contoured symbols represent episodes where fever ceased, while shaded symbols represent episodes with present or recurrent fever refractory to broad spectrum antibiotic treatment. Small sized symbols represent children. Numbers intend to denote patient ID numbers. [file 12879_2015_995_MOESM2_ESM.docx]

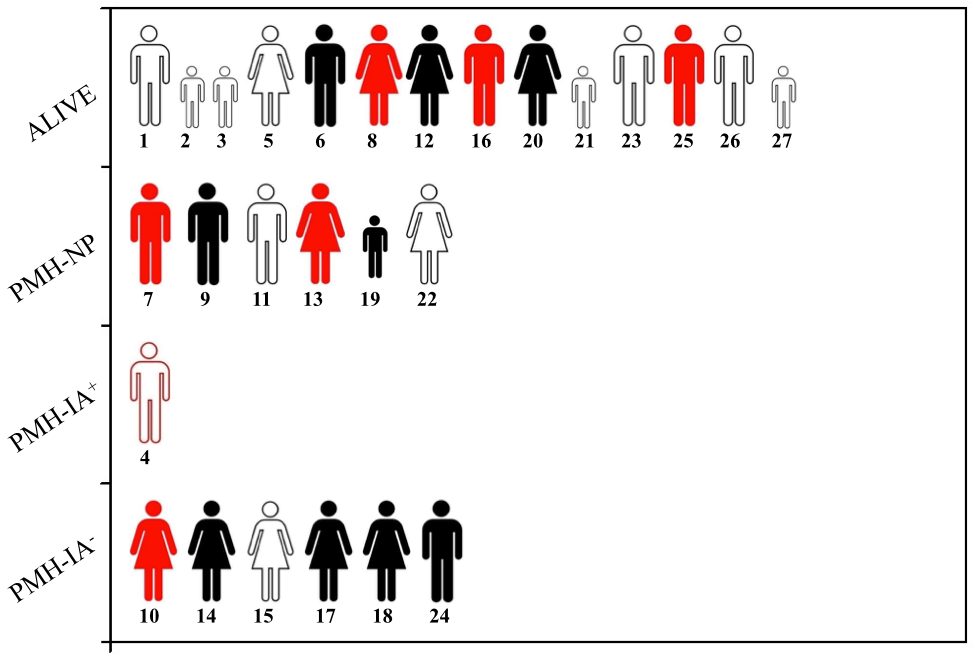


**Figure S2**

**Representation of the data on postmortem histology**

(IA^+^) the presence of hyphae representative of invasive *fungal* infection was confirmed by PAS or H&E staining. (IA^-^) the presence of hyphae could not be confirmed by PAS or H&E staining. Black symbols represent controls while red symbols represent cases. Contoured symbols represent episodes where fever ceased, while shaded symbols represent episodes with present or recurrent fever refractory to broad spectrum antibiotic treatment. Small sized symbols represent children. Numbers intend to denote patient ID numbers.
