# Supplementary material for: Combining standard clinical methods with PCR showed improved diagnosis of invasive pulmonary aspergillosis in patients with hematological malignancies and prolonged neutropenia
Source: BMC Infect Dis. 2015 Jul 1;15:251. doi: 10.1186/s12879-015-0995-8 (PMC4487853; doi:10.1186/s12879-015-0995-8)
Supplement: Additional file 3: Figure S3. — Postmortem histological findings from case ID 4 showing congestion and focal atelectasis of the lung (3A, ×10 magnification, H&E staining). Detailed evaluation underlined the Aspergillus specific PCR assay based assumption of the presence of invasive Aspergillus hyphae (3B). This was also supported by PAS staining (3C, ×40 magnification) although definite identification is not possible on a morphological basis therefore the presence of other filamentous fungi cannot be excluded. [file 12879_2015_995_MOESM3_ESM.docx]

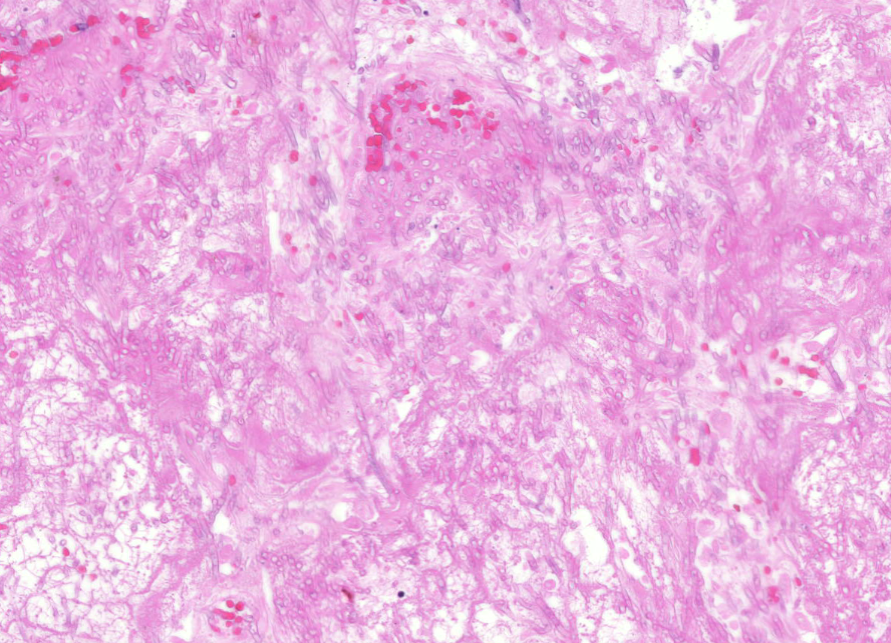

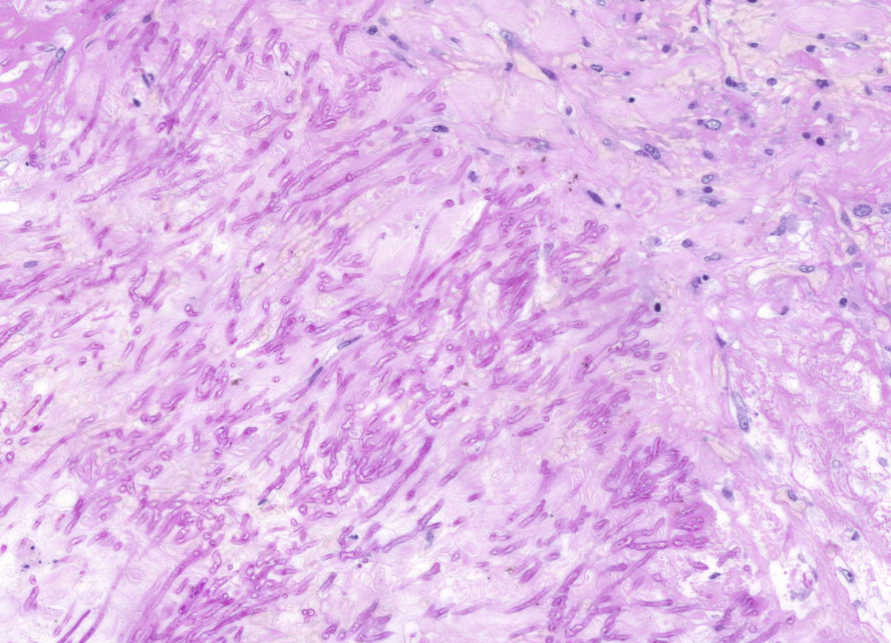

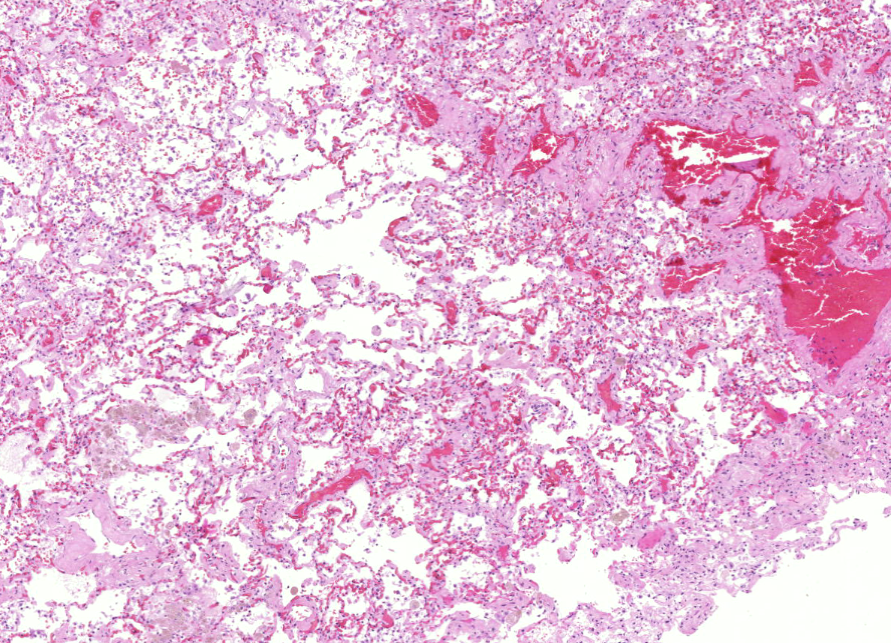
**Figure S3**

**Figure S3/C**

**Figure S3/B**

**Figure S3/A**

Postmortem histological findings from case ID 4 showing congestion and focal atelectasis of the lung (3A, x10 magnification, H&E staining). Detailed evaluation underlined the *Aspergillus* specific PCR assay based assumption of the presence of invasive *Aspergillus* hyphae (3B). This was also supported by PAS staining (3C, x40 magnification) although definite identification is not possible on a morphological basis therefore the presence of other filamentous fungi cannot be excluded.
