# Supplementary material for: Combining standard clinical methods with PCR showed improved diagnosis of invasive pulmonary aspergillosis in patients with hematological malignancies and prolonged neutropenia
Source: BMC Infect Dis. 2015 Jul 1;15:251. doi: 10.1186/s12879-015-0995-8 (PMC4487853; doi:10.1186/s12879-015-0995-8)
Supplement: Additional file 4: Figure S4. — Gross view (4/A, ×10 magnification) and high-power image (4/B, ×40 magnification) of the postmortem lung specimens in case ID 24. Atelectasis with fibrinohemorrhagic exudate and alveolar damage is clearly visible but evidence of microscopic fungal manifestation is absent. [file 12879_2015_995_MOESM4_ESM.docx]

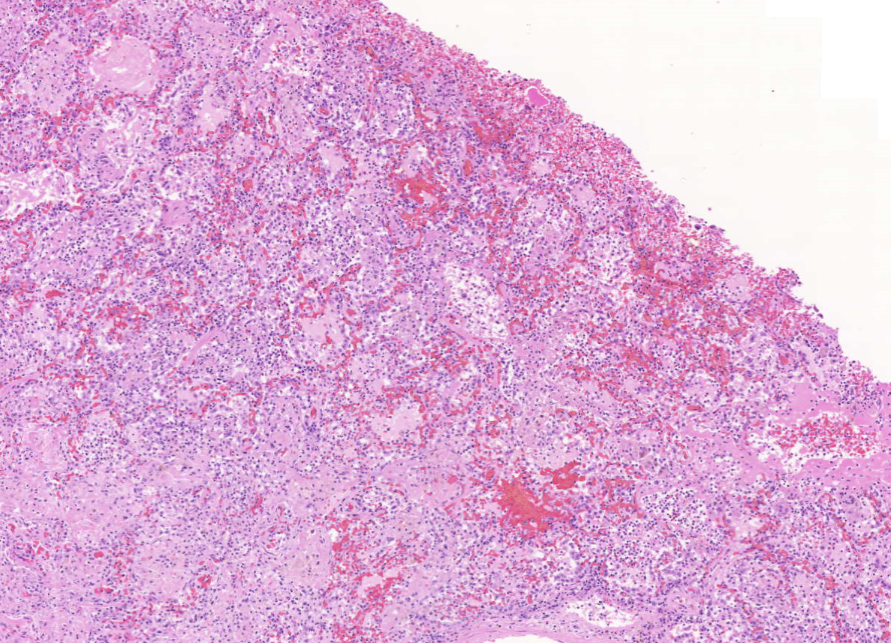

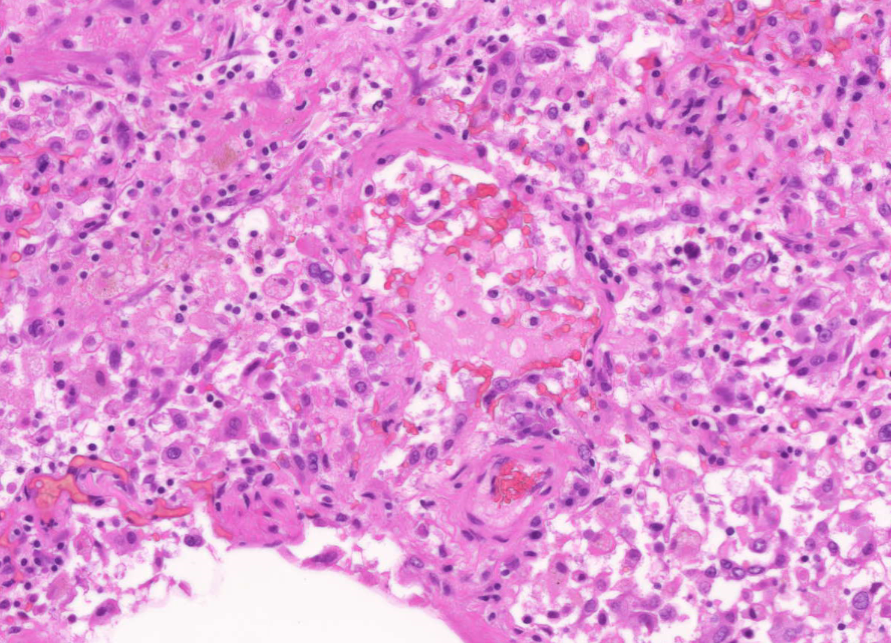


**Figure S4.** Gross view (**4/A**, x10 magnification) and high-power image (**4/B**, x40 magnification) of the postmortem lung specimens in case ID 24. Atelectasis with fibrinohemorrhagic exudate and alveolar damage is clearly visible but evidence of microscopic fungal manifestation is absent.

**Figure S4/A**

**Figure S4/B**
